# SARS-CoV-2 Mutations and Variants May Muddle the Sensitivity of COVID-19 Diagnostic Assays

**DOI:** 10.3390/microorganisms10081559

**Published:** 2022-08-02

**Authors:** Mohammad Alkhatib, Luca Carioti, Stefano D’Anna, Francesca Ceccherini-Silberstein, Valentina Svicher, Romina Salpini

**Affiliations:** 1 Department of Experimental Medicine, University of Rome “Tor Vergata”, 00133 Rome, Italy; mohammad--alkhatib@hotmail.com (M.A.); luca.carioti@yahoo.com (L.C.); stefanodanna26@gmail.com (S.D.); ceccherini@med.uniroma2.it (F.C.-S.); valentina.svicher@uniroma2.it (V.S.); 2 Department of Biology, University of Rome “Tor Vergata”, 00133 Rome, Italy

**Keywords:** COVID-19, SARS-CoV-2, variants, mutations, PCR, diagnostic-escape, primer-mismatches

## Abstract

The performance of diagnostic polymerase chain reaction (PCR) assays can be impacted by SARS-CoV-2 variability as this is dependent on the full complementarity between PCR primers/probes and viral target templates. Here, we investigate the genetic variability of SARS-CoV-2 regions recognized by primers/probes utilized by PCR diagnostic assays based on nucleotide mismatching analysis. We evaluated the genetic variation in the binding regions of 73 primers/probes targeting the Nucleocapsid (N, N = 36), Spike (S, N = 22), and RNA-dependent RNA-polymerase/Helicase (RdRp/Hel, N = 15) of the publicly available PCR-based assays. Over 4.9 million high-quality SARS-CoV-2 genome sequences were retrieved from GISAID and were divided into group-A (all except Omicron, >4.2 million) and group-B (only Omicron, >558 thousand). In group-A sequences, a large range of variability in primers/probes binding regions in most PCR assays was observed. Particularly, 87.7% (64/73) of primers/probes displayed ≥1 mismatch with their viral targets, while 8.2% (6/73) contained ≥2 mismatches and 2.7% (2/73) contained ≥3 mismatches. In group-B sequences, 32.9% (24/73) of primers/probes were characterized by ≥1 mismatch, 13.7% (10/73) by ≥2 mismatches, and 5.5% (4/73) by ≥3 mismatches. The high rate of single and multiple mismatches- found in the target regions of molecular assays used worldwide for SARS-CoV-2 diagnosis reinforces the need to optimize and constantly update these assays according to SARS-CoV-2 genetic evolution and the future emergence of novel variants.

## 1. Introduction

The coronavirus disease 2019 (COVID-19) pandemic has recorded over 554 million cases of infection (about 7% of the globe) and is responsible for more than 6.3 million deaths in the last 30 months. The causative agent of COVID-19; SARS-CoV-2 (severe acute respiratory syndrome coronavirus 2) is characterized by an RNA genome encoding more than 29 structural, non-structural, and regulatory proteins.

As an RNA virus, SARS-CoV-2 has a relatively low dynamic mutation rate compared to other RNA viruses, including influenza, HIV, and HCV, and even to DNA viruses such as HBV [1], mainly due to the transcriptional fidelity and proofreading activity of its replication complex. Despite that, since the emergence of SARS-CoV-2, adaptive evolution and genetic diversification have led to the emergence of over 56,000 mutations, including deletions and insertions across the viral genome. In particular, most mutations are observed in the ORF1ab of 71.3%, followed by 12.8% in the Spike and 4.2% in the Nucleocapsid, [2] (Accessed date, 3 July 2022). 

To date, SARS-CoV-2 genetic diversification has led to the emergence of the variants of concern (VOC), including Alpha, Beta, Gamma, Delta, and more recently Omicron. These variants have raised several concerns due to their potential impact on increasing transmissibility and severity [3,4]. Therefore, efficient surveillance and accurate detection of SARS-CoV-2 variants are crucial to optimize clinical management and effective pandemic control. This also requires accurate and reliable diagnostic molecular assays based on the Polymerase chain reaction (PCR) as well as antigen-based (Ag-RDT) assays, as highlighted by the WHO, CDC, and ECDC [5,6,7].

Notably, the performance of the diagnostic assays can be impacted by certain variants. As the performance of PCR assays is crucially dependent on the set of primers and probes specific to bind complementary sequences in the targeted viral genome, mismatches between primers and templates are known to influence assay efficiency and sensitivity [8,9,10,11,12,13,14]. Notably, most of the validated SARS-CoV-2 PCR assays, including those for real-time (RT)-PCR, Qualitative PCR, and Sequencing, had their set of primers and probes designed based on the wide-type strain (NC_045512.2) published by early January 2020 and targeted regions in the Nucleocapsid, Spike, Envelope, and ORF1ab. 

As above stated, these targeted regions have the highest mutational rates; therefore, some mutations may occur in the binding regions of the primers or probes, potentially leading to primer/probe-template mismatches and, consequently, false-negative results or even detection failure. Herein, the objective was to evaluate the genetic variability of the viral regions recognized by primers and probes utilized by the publicly available PCR-based diagnostic assays based on nucleotide mismatching analysis.

## 2. Materials and Methods

In this study, SARS-CoV-2 genome sequences (N = 4,930,239, Accessed date 25 January 2022) were retrieved from the Global Initiative on Sharing All Influenza Data (GISAID) database [15], and about half of the sequences were from Europe. Stringent quality filters were applied to include only entire sequences characterized by high quality (identified, for each analyzed region, the presence of <1% ambiguous nucleotides, <0.05% unique amino acid mutations, and no insertion/deletion unless verified in the sequence by the submitter).

We aligned the sequences using Bioedit software and the MAFFT server against the reference sequence (NC_045512.2). Subsequently, we marked, in the alignments, the binding sites of 73 primers/probes targeting the Nucleocapsid (N, N = 36), Spike (S, N = 22), and RNA-dependent RNA-polymerase/Helicase (RdRp/Hel, N = 15) of the publicly available (reported by the WHO and/or published articles) PCR as RT-PCR, Qualitative PCR, and Sequencing assays used for detection and characterization of SARS-CoV-2 [16,17,18,19,20,21,22].

The sequences were divided into group A (all sequences except Omicron) and group B (only Omicron sequences). The frequency of mismatches was calculated based on a total of 4,133,465 sequences for Nucleocapsid, 4,196,498 Spike glycoprotein, and 4,132,890 RdRp and Helicase for all SARS-CoV-2 sequences apart from Omicron (Group A) and for 558,914 sequences of Nucleocapsid, Spike glycoprotein, RdRp, and Helicase for predominately circulating Omicron VOC (Group B).

We then calculated the frequency of at least one, two, and three mismatches in the binding region of each primer and probe targeting the Nucleocapsid, Spike, RNA-dependent RNA polymerase, and Helicase. Sequences with a mixture of wild-type and mutant residues at single positions were considered to have the mutation(s) at that position. In particular, we calculated the number of exact matches, zero errors, and mismatches, with one, two or three errors, searching for this in the two groups of sequences using the software tre-agrep, a software that allow to search a string in a file with approximate matches. To standardize the effect of the sequence, only primers/probes mismatches observed in >1% of viral sequences (corresponding to >41,000 group-A and >5500 group-B sequences) were considered.

## 3. Results and Discussion

The overall analysis revealed that in group-A sequences, which represent the overall dataset of sequences except for the Omicron, a large range of variability in primers/probes binding regions was observed in most PCR assays. Particularly, 87.7% (64/73) of primers/probes displayed ≥1 mismatch with their viral targets, while 8.2% (6/73) displayed ≥2 mismatches and 2.7% (2/73) ≥3 mismatches (Table 1). Whereas, in group-B sequences (Omicron alone), 32.9% (24/73) of primers/probes was characterized by ≥1 mismatch, 13.7% (10/73) by ≥2 mismatches, and 5.5% (4/73) by ≥3 mismatches. (Table 1).

It is widely known that viruses tend to evolve rapidly during outbreaks, leading to emerging new mutations, and it is unsurprising that mutations either synonymous or non-synonymous may occur in the binding regions of primers and probes compromising the sensitivity of PCR assays.

Focusing on group A, most primers/probes with ≥1 mismatch target the N gene (97.2%; 35/36), followed by the S (86.4%; 19/22), and the RdRp/Hel (66.7%; 10/15). The six primers/probes with ≥2 mismatches target mostly the N gene (8.3%; 3/36), and then the S (9.1%; 2/22), and the RdRp/Hel (6.7%; 1/15). Importantly, the two primers displaying ≥3 mismatches target the S and N genes. Notably, the highest number of mismatches was observed in the N forward primer of RT-PCR, designed by CDC in China, where 83.3%, 34.6%, and 33.8% of group A sequences displayed ≥1, ≥2, and ≥3 mismatches, respectively. 

This was due to the three-nucleotide substitutions codifying the mutation pair R203K/G204R that is present in several SARS-CoV-2 variants, including previous VOCs Alpha and Gamma. The specific mutations R203M for Delta and T205I for Beta also localize in this primer as well, thus explaining the high frequency of mismatching for this primer (Table 2). 

Our finding is in line with an earlier study by Vogels et al. that showed the three mismatches in the China CDC N forward primer caused by the R203K/G204R [23]. Likewise, in the spike, in the RT-PCR forward primer reported by Young et al. in Singapore, ≥1, ≥2, and ≥3 mismatches were observed in 38.6%, 23.2%, and 22.1% of the sequences, respectively, the reason was attributable to the six-nucleotide deletions leading to H69del-V70del. The latter was associated with diagnostic escape events termed S gene target failure or S gene dropout in previously Alpha VOC [24,25] (Table 1 and Table 2).

Moreover, some primers/probes have shown various degrees of mismatches arriving at 73%, as the highest mismatches of ≥1, observed in both S overlapping probes from RT-PCR by Sigma-Aldrich. This can be attributed to the mutation’s enrichment in this region (Q677H, N679K, Ins679GIAL, P681H, and P681R) that characterize variants, including Alpha, Gamma, and Delta VOCs. 

This was followed by an S forward primer designed by Thermo Fisher, which showed ≥1 and ≥2 mismatches of 48.7% and 3.2% of sequences, respectively, resulting from the mutations L18F, T19R, and T20N that characterize Beta, Gamma, and Delta VOCs. Similarly, the two RdRp forward primers designed by Charité Hospital and Won from Korea were observed with about 42% of ≥1 mismatch due to the Delta VOC mutation G671S and other sporadic mutations M666I and M666T found in other variants. 

Moreover, the N overlapping reverse primers for qualitative PCR, designed by Sigma-Aldrich, have shown ≥1 mismatch in binding sequences in over 37% of sequences, and this could be due to the localization of the reverse complement in a region highly enriched in mutations, including G215C specific for Delta VOC, and other sporadic mutations (Table 1 and Table 2). 

Again, in the same assay from CDC in China, the N reverse primer of RT-PCR showed ≥1 mismatch in 32.7% of sequences, and this is due to the presence of S235F specific for Alpha VOC and M234I in different SARS-CoV-2 lineages. Finally, in the RT-PCR probe reported by Young et al. in Singapore, ≥1 mismatch was observed in 13% of the sequences, due to the presence of D80A in Beta VOC and K77T in some sublineages of Delta VOC (Table 1 and Table 2).

In three RT-PCR assays (one of which is the N probe reported by Young et al. in Singapore, the second of which is the Hel reverse primer reported by Chan et al., and the last is the RdRp reverse primer designed by Charite hospital), the primers/probe showed ≥1 mismatch for all the sequences, due to the original nucleotide mismatching (designed incorrectly) to the SARS-CoV-2 wide-type sequence as highlighted in Table 1. Focusing on RdRp reverse primer by Charite hospital (the first assay that has been broadly criticized for its molecular and methodological validities), it has been demonstrated that a single base mismatch in the reverse primer can increase the number of quantification cycles and reduce the sensitivity of the assay by affecting the RT step [26].

Similar to group A, in group B sequences, most primers/probes with ≥1 mismatch target the N gene (30.6%; 11/36), followed by the S gene (36.4%; 8/22), and RdRp/Hel (33.3%; 5/15). The 10 primers/probes with ≥2 mismatches target mostly the S (22.7%; 5/22), followed by N (8.3; 3/36), and RdRp/Hel (13.3%; 2/15). Importantly, ≥3 mismatches were found in four primers targeting the S and N genes, thus, representing the highest number of mismatches (Table 1).

Similar to group A sequences, the highest number of mismatches was observed in the N forward primer of RT-PCR, designed by CDC in China, where >99% of sequences showed ≥1, ≥2, and ≥3 mismatches, due to the presence of the mutation pair R203K/G204R in all Omicron sublineages (Table 1 and Table 2). Likewise, it was observed in the N reverse primer of RT-PCR assay, designed by NIH in Thailand, that >99% of sequences showed ≥3 mismatches, due to the presence of the nine nucleotide deletion codified as E31-R32-S33 in all Omicron sublineages (Table 1 and Table 2). 

In the S forward primer of the RT-PCR assay reported by Young et al., ≥3 mismatches were observed in >97% of the sequences, again due to the presence of the deletion at H69del-V70del in the Omicron’s backbone except for BA.2 sublineage. Furthermore, the S forward primer designed by Thermo Fisher, showed ≥1, ≥2, and ≥3 mismatches, of 4.2%, 2.2%, and 2.2% of sequences, respectively, resulting from the mutations T19R, L24S, and P25del that characterize BA.2 sublineage of Omicron VOC (Table 1 and Table 2).

Other primers/probes have shown complete (100%) mismatches, and the highest mismatches were observed again in both S overlapping probes of RT-PCR assay by Sigma-Aldrich of ≥1 and ≥2 mismatches, due to the presence of the mutations N679K and P681H in all Omicron VOC sublineages. A similar scenario was observed for the N probe of RT-PCR assay designed by CDC in the USA, which showed ≥1 mismatch in >99.7% of Omicron sequences due to the presence of P13L mutation. Again, the S probe reported by Chan et al. showed ≥1 mismatch in 97.2% of sequences, because of the existence of the mutation K417N in Omicron’s backbone (Table 1 and Table 2). In the S reverse primer of Qualitative PCR reported by Won et al. in Korea, >97.4% of Omicron sequences showed ≥1 mismatch with the reverse primer due to the presence of the T547K mutation (Table 1 and Table 2).

Notably, the first study that addressed this issue has been published in May 2020 as early as the pandemic began [27] and showed that 79% (26/33) of the primer binding sites used in the RT-PCR assays (including CDC China and CDC USA assays) were mutated in at least one genome sequence of the total 1825 analyzed SARS-CoV-2 sequences.

Soon afterward, a number of studies described impairment of detection due to primer or probe mismatches. Artesi and colleagues showed that a single nucleotide mutation (C to T) at position 96 in the E gene is associated with failure of the Cobas SARS-CoV-2 E gene RT-PCR [28]. A study from our group also reported the failure of N target detection due to a deletion of six nucleotides at position 640–645 in the N gene of AllplexTM SARS-CoV2 Assay [29]. 

Three nucleotides’ mismatches at N-tail of N gene lead to D3L mutation in the previously Alpha VOC is associated with N gene dropout and CT value shifting to Allplex™ SARS-CoV-2/FluA/FluB/RSV™ PCR assay [30]. Furthermore, a PCR amplification curve abnormality (double or low amplification curve) was reported in the RdRp/S gene of the Allplex SARS-CoV-2 assay due to the spike mutation in the P681 of Alpha and Delta VOCs [31]. This could be helpful in rapidly predicting the presence of these variants prior to sequencing. 

The single nucleotide polymorphism C to T at position 927 in the C terminus of the N gene has been reported to cause N gene target failure for an Xpert Xpress SARS-CoV-2 (GXP) assay [32,33]. Finally, a single point substitution G to T at 922 was sufficient to impair N gene detection in Cepheid Xpert Xpress SARS-CoV-2 assay as recently reported in a Singaporean study [34]. This substitution localized in a region targeted by the N2 probe from CDC USA assay, and we observed that 2.1% of group A sequences presented ≥1 mismatch and <0.4% of group B in Omicron sequences due to the lack of mutation in the targeted region.

It is important to note that each mismatch, irrespective of its location within the primer sequence, leads to the reduced thermal stability of the primer-template duplex, thus potentially affecting the PCR performance. However, mismatches located in the 3′ end region of a primer have significantly larger effects on the priming efficiency compared with 5′ located mismatches, since 3′ end mismatches can disrupt the nearby polymerase active site [8]. As seen in Table 2, the mutations responsible for the most mismatches in primer/probe-template resided more frequently in the 3′ end or both ends.

Overall, the sensitivity of PCR assays in detecting SARS-CoV-2 is highly dependent on the virus’ genetic variability, which can be determined by matching the primer and probe to sequence binding region. Interestingly, currently, multi-gene target PCR assays are considered the gold standard for the detection of SARS-CoV-2; thereby, a single gene detection failure does not jeopardize the proper interpretation in multiplex assays targeting different genes of the viral genome. The detection of mutations that may have the potential to escape diagnostic assay is a must for eliminating any resulting discrepancy and better interpretation. It is important to note that the detection of SARS-CoV-2 with one gene detection failure or dropout may provide a rapid signal that a specific variant may be present; thus, sequencing can be considered to characterize the variant.

Unlike PCR assays, the sensitivity of Ag-RDT assays is partially dependent on SARS-CoV-2 variants and mutation presence. In a recently published study that compared the sensitivity of seven Ag-RDT assays between all VOCs, including Omicron, the results showed that the analytical sensitivity to detect the Omicron variant was lower than that for the other VOCs in most of the assays evaluated [35]. Thus, potentially due to the presence of mutations and deletions that Omicron possesses in the nucleocapsid, which is the target of nearly all Ag-RDT assays. 

A multi-center study compared another seven Ag-RDT assays, regardless of the SARS-CoV-2 variants, found that all Ag-RDTs reach high sensitivity early in the disease (<3 days of symptoms) and in individuals with high viral loads (>6 log10 SARS-CoV2 RNA copies/mL) irrespective of whether symptomatic or asymptomatic cases [36]. Inline, another study demonstrated increasing the Ag-RDT assay’s sensitivity when the viral load (≥5.2 log10 SARS-CoV2 E gene RNA copies/mL) and comparable performance between symptomatic and asymptomatic cases with similar viral loads [37]. 

A recent study showed that the nucleocapsid mutation T135I was associated with escaping detection by the Panbio COVID-19 rapid antigen test due to its localization around major epitopes of N protein [38]. The latest study from the USA compared the performance of three Ag-RDT assays in the detection of Delta and Omicron VOC, the results showed that the Ag-RDT assays performed similarly for Omicron and Delta VOCs and performed better among patients with the highest viral loads [39].

The overall findings suggest that the sensitivity of Ag-RDT assays is widely dependent on several factors and is not only restricted to viral load or symptoms status nonetheless it may also be extended to mutations that can possibly alter different epitopes of SARS-CoV-2 structural proteins leading to escape coated antibodies in Ag-RDT assays. It is noteworthy to mention that low viral load (high CT) and mutations may pose a challenge in the early detection of SARS-CoV-2 by Ag-RDT assays, thus, further fueling chain transmission.

Importantly, our results are in keeping with the FDA recommendations for the use of assays with multiple genetic targets ensuring higher sensitivity despite the different genetic profiles of SARS-CoV-2 variants. Multiple genetic targets implies that a molecular assay is designed to detect more than one region of the SARS-CoV-2 genome or, for antigen tests, more than one region of the proteins that form SARS-CoV-2 [40]. Furthermore, assay optimization and validation are essential to confirm its sensitivity and specificity, and thus can be ensured by designing homologous primers to the target sequence, verifying the reverse complement, avoiding ambiguous nucleotides unless necessary, and optimizing the primers’ concentration and temperature.

## 4. Conclusions

This study highlights the importance of characterizing mutations and variants of SARS-CoV-2 as they have the potential to affect diagnostic assays. The high rate of single and multiple mismatches found in the target regions of molecular assays worldwide used for SARS-CoV-2 diagnosis reinforces the need to use more than one target to bypass the potential lack of recognition of one PCR target and to monitor and constantly update, if necessary, these assays according to SARS-CoV-2 genetic evolution and the future emergence of novel variants. This will ensure the full efficacy of diagnostic assays, thus, contributing to the goal of limiting viral transmission chains and contrasting viral spread.

## Figures and Tables

**Table 1:** Summary of primers and probes mismatches of RT-PCR, Qualitative PCR, and Sequencing assays posted by WHO and published by original articles for detection of SARS-CoV-2.

Assay ^a^	Gene ^b^	Primer/Probe Sequence	Primer	Start ^c^	End ^c^	Mismatch Targets in Genomes ^d^ (Frequency)			Assay Reference
						>1	>2	>3	
						***Frequencies in Group A/B***			
***Real-time PCR primers***									
CDC China	N	GGGGAACTTCTCCTGCTAGAAT	F	608	629	**83.25/99.90**	**34.60/99.17**	**33.76/99.15**	[20]
	N	CAGACATTTTGCTCTCAAGCTG	R	552	573	**32.73**/0.65	0.42/0.08	0.05/0.03	
	N	TTGCTGCTGCTTGACAGATT	P	661	680	**1.59**/0.64	0.03/0.43	0.02/0.42	
Charité Hospital Germany	N	CACATTGGCACCCGCAATC	F	433	451	**1.02**/0.36	0.01/0.01	0.00/0.01	[17,20]
	N	GAGGAACGAGAAGAGGCTTG	R	698	717	**2.12**/0.64	0.04/0.03	0.01/0.01	
	N	ACTTCCTCAAGGAACAACATTGCCA	P	480	504	**1.57**/0.65	0.04/0.03	0.02/0.01	
CDC USA	N1	GACCCCAAAATCAGCGAAAT	F	14	33	**3.96/3.18**	0.02/0.02	0.00/0.00	[20]
	N1	TCTGGTTACTGCCAGTTGAATCTG	R	1173	1196	**1.20**/0.21	0.01/0.05	0.01/0.01	
	N1	ACCCCGCATTACGTTTGGTGGACC	P	36	59	**3.08/99.79**	0.06/0.40	0.01/0.01	
	N2	TTACAAACATTGGCCGCAAA	F	891	910	**1.78**/0.28	0.02/0.01	0.01/0.00	
	N2	GCGCGACATTCCGAAGAA	R	301	318	**1.48**/0.34	0.02/0.01	0.01/0.00	
	N2	ACAATTTGCCCCCAGCGCTTCAG	P	915	937	**2.12**/0.36	0.04/0.02	0.01/0.00	
	N3	GGGAGCCTTGAATACACCAAAA	F	408	429	**1.81/2.15**	0.02/0.04	0.01/0.02	
	N3	TGTAGCACGATTGCAGCATTG	R	779	799	**1.11**/0.32	0.03/0.01	0.01/0.01	
	N3	AYCACATTGGCACCCGCAATCCTG	P	431	454	**1.61**/0.43	0.02/0.27	0.00/0.02	
NIID Japan	N	AAATTTTGGGGACCAGGAAC	F	852	871	**1.20**/0.25	0.03/0.01	0.01/0.01	[20]
	N	TGGCACCTGTGTAGGTCAAC	R	249	268	**1.86**/0.40	0.05/0.02	0.02/0.01	
	N	ATGTCGCGCATTGGCATGGA	P	949	968	**1.46**/0.36	0.04/0.04	0.02/0.03	
HKU Med Hong-Kong	N	TAATCAGACAAGGAACTGATTA	F	892	893	**1.23**/0.27	0.02/0.01	0.01/0.00	[20]
	N	CGAAGGTGTGACTTCCATG	R	277	295	**2.70**/0.44	0.04/0.01	0.01/0.01	
	N	GCAAATTGTGCAATTTGCGG	P ^e^	335	354	**1.65**/0.25	0.04/0.01	0.01/0.00	
NIH Thailand	N	CGTTTGGTGGACCCTCAGAT	F	47	66	**1.86/3.15**	0.02/0.01	0.00/0.00	[20]
	N	CCCCACTGCGTTCTCCATT	R	1155	1173	**1.34/99.49**	0.05/**99.45**	0.01/**99.39**	
	N	CAACTGGCAGTAACCA	P	67	84	0.96/**3.11**	0.01/0.05	0.00/0.03	
Chan China	N	GCGTTCTTCGGAATGTCG	F	937	954	**1.54**/0.33	0.02/0.01	0.01/0.00	[16]
	N	TTGGATCTTTGTCATCCAATTTG	R	225	247	**1.56/13.04**	0.02/0.06	0.01/0.01	
	N	AACGTGGTTGACCTACACAGST	P	984	1005	**2.08/1.79**	**1.20**/0.16	0.01/0.01	
Young Singapore	N	CTCAGTCCAAGATGGTATTTCT	F	310	331	**1.40/2.94**	0.01/0.01	0.00/0.00	[22]
	N	AGCACCATAGGGAAGTCC	R	883	900	**1.01**/0.12	0.02/0.02	0.01/0.01	
	N	ACCTAGGAACTGGCCCAGAAGCT	P	335	357	**100/100**	**1.26/2.89**	0.02/0.02	
Young Singapore	S	TATACATGTCTCTGGGACCA	F	201	220	**38.59/97.37**	**23.20/97.03**	**22.08/97.01**	[22]
	S	ATCCAGCCTCTTATTATGTTAGAC	R	3506	3529	**1.51/1.68**	0.11/0.02	0.04/0.01	
	S	CTAAGAGGTTTGATAACCCTGTCCTACC	P	227	254	**12.96/2.51**	0.28/0.36	0.18/0.31	
Chan China	S	CCTACTAAATTAAATGATCTCTGCTTTACT	F	1150	1179	**1.92**/0.32	0.06/0.16	0.03/0.09	[16]
	S	CAAGCTATAACGCAGCCTGTA	R	2513	2533	**1.81**/0.58	0.03/0.39	0.01/0.24	
	S	CGCTCCAGGGCAAACTGGAAAG	P	1230	1251	**6.43/97.22**	0.07/2.5	0.01/0.37	
Sigma-Aldrich	S1	CAGGTATATGCGCTAGTTATCAGAC	F	2003	2027	**1.79**/0.37	0.02/0.02	0.01/0.01	[18]
	S1	CCAAGTGACATAGTGTAGGCAATG	R	1721	1744	**2.00**/0.26	0.02/0.14	0.01/0.08	
	S1	AGACTAATTCTCCTCGGCGGGCACG	P	2030	2054	**73.34/99.97**	0.90/**91.34**	0.02/0.16	
	S2	GCAGGTATATGCGCTAGTTATCAG	F	2002	2025	**1.73**/0.35	0.02/0.01	0.01/0.01	
	S2	ACACTGGTAGAATTTCTGTGGTAAC	R	1632	1656	**1.00**/0.23	0.10/0.11	0.07/0.05	
	S2	CTAATTCTCCTCGGCGGGCACG	P	2033	2054	**72.42/99.97**	0.55/**91.34**	0.02/0.12	
Charité Hospital Germany	RdRp1	GTGARATGGTCATGTGTGGCGG	F	1991	2012	**42.34**/0.34	0.14/0.01	0.00/0.00	[17,20]
	RdRp1	CARATGTTAAASACACTATTAGCATA	R	707	732	**100/100**	0.11/2.38	0.01/0.05	
	RdRp1	CCAGGTGGWACRTCATCMGGTGATGC	P1	2029	2054	**1.11**/0.11	0.06/0.05	0.05/0.02	
	RdRp1	CAGGTGGAACCTCATCAGGAGATGC	P2	2030	2054	**1.14/**0.11	0.06/0.05	0.05/0.02	
Institut Pasteur France	RdRp-IP4	GGTAACTGGTATGATTTCG	F	640	658	0.41/0.18	0.17/0.08	0.16/0.08	[20]
	RdRp-IP4	CTGGTCAAGGTTAATATAGG	R	2051	2070	0.98/0.48	0.01/0.02	0.00/0.01	
	RdRp-IP4	TCATACAAACCACGCCAGG	P	665	683	**4.77/1.53**	0.03/0.03	0.01/0.03	
Chan China ^f^	RdRp/Hel	CGCATACAGTCTTRCAGGCT	F	2780/1	2796/3	**2.16/1.14**	0.04/0.98	0.02/0.97	[16]
	Hel	GTGTGATGTTGAWATGACATGGTC	R	1687	1710	**100/100**	**2.68/1.25**	0.57/0.19	
	Hel	TTAAGATGTGGTGCTTGCATACGTAGAC	p	40	67	**2.66/1.34**	0.05/0.03	0.02/0.01	
Young Singapore	RdRp	TCATTGTTAATGCCTATATTAACC	F	715	738	0.58/0.37	0.01/0.01	0.00/0.00	[22]
	RdRp	CACTTAATGTAAGGCTTTGTTAAG	R	1994	2017	0.81/0.42	0.02/0.01	0.01/0.00	
	RdRp	AACTGCAGAGTCACATGTTGACA	P	753	775	0.80/0.34	0.02/0.01	0.01/0.01	
***Quantitative PCR and Sequencing primers***									
Won Korea	N	CAATGCTGCAATCGTGCTAC	F	459	478	**1.10**/0.32	0.03/0.01	0.00/0.01	[21]
	N	GTTGCGACTACGTGATGAGG	R	682	701	**2.31**/0.57	0.03/0.02	0.01/0.01	
Sigma-Aldrich	N1	GCCTCTTCTCGTTCCTCATCAC	F	544	565	**2.17**/0.52	0.03/0.02	0.01/0.01	[18]
	N1	AGCAGCATCACCGCCATTG	R	604	622	**36.93**/0.71	0.34/0.04	0.02/0.03	
	N2	AGCCTCTTCTCGTTCCTCATCAC	F	543	565	**2.18**/0.64	0.03/0.02	0.01/0.01	
	N2	CCGCCATTGCCAGCCATTC	R	614	632	**37.69**/0.74	0.37/0.03	0.07/0.01	
Won Korea	S	CTACATGCACCAGCAACTGT	F	1552	1571	0.79/0.60	0.12/0.25	0.04/0.09	[21]
	S	CACCTGTGCCTGTTAAACCA	R	2169	2188	0.66/**97.46**	0.01/0.04	0.00/0.01	
NIID Japan	S1	TTGGCAAAATTCAAGACTCACTTT	F	2792	2815	**1.76**/0.71	0.02/0.08	0.01/0.01	[20]
	S1	TGTGGTTCATAAAAATTCCTTTGTG	R	482	506	**2.02**/0.29	0.01/0.01	0.00/0.00	
	S2	TCAAGACTCACTTTCTTCCAC	F	2802	2822	**1.28**/0.26	0.10/0.02	0.01/0.01	
	S2	ATTTGAAACAAAGACACCTTCAC	R	526	548	**1.96**/0.22	0.03/0.03	0.00/0.01	
	S	AAGACTCACTTTCTTCCACAG	F	2804	2824	**1.32**/0.29	0.10/0.03	0.01/0.02	
	S	CAAAGACACCTTCACGAGG	R	534	552	**1.97**/0.20	0.13/0.04	0.00/0.01	
Thermo Fisher	S	GTGTTAATCTTACAACCAGAACTCAATTAC	F	44	73	**48.73/4.16**	**3.22/2.18**	0.05/2.18	[19]
	S	CACAGACTTTAATAACAACATTAGTAGCG	R	3426	3454	0.48/0.11	0.13/0.04	0.06/0.01	
Won Korea	RdRp	CATGTGTGGCGGTTCACTAT	F	2001	2020	**42.21**/0.24	0.11/0.05	0.03/0.03	[21]
	RdRp	TGCATTAACATTGGCCGTGA	R	679	698	**1.08**/0.18	0.01/0.09	0.01/0.08	

The overall number of analyzed sequences in group A was 4,133,465 for Nucleocapsid, 4,196,498 for Spike, and 4,132,890 for RdRp and Helicase, and in group B was 558,914 for Nucleocapsid, 558,914 for Spike, and 558,914 for RdRp and Helicase. The mismatch was defined as at least one nucleotide mutation observed in the primer sequence, and mismatches of over 1% are highlighted in **bold**. ^a^ Assay names are reported exactly as they were named in their references. ^b^ Only assays that targeted the Nucleocapsid, Spike, RdRp, and Helicase are reported. ^c^ Numbering was set for each protein nucleotide: 1-1257 for Nucleocapsid, 1-3819 for Spike, 1-2796 for RdRp, and 1-1803 for Helicase. ^d^ Mismatch target is the disagreement between the expected target nucleotide and the nucleotide in the genome (*reported as frequencies in group-A/group-B*). ^e^ The probe was designed based on a reverse complement. ^f^ The only assay herein that targets two consequent proteins, the forward primer starts in the last 17 nucleotides in RdRp and ends in the first three nucleotides in helicase, while both the reverse primer and probe start and end in Hel. Abbreviations: F, forward primer; R, reverse primer; P, probe; N, nucleocapsid; S, spike; RdRp, polymerase; and Hel, helicase.

**Table 2:** Summary of SARA-CoV-2 specific variants mutations located in the primers/probe.

Assays	Protein	Primers/Probes	Direction	Mutation Location	SARS-CoV-2 Varaints					
RT-PCR Assays					Alpha	Beta	Gamma	Delta	Omicron	Other Variants
China CDC	N	GGGGAACTTCTCCTGCTAGAAT	F	5′ end	R203K, G204R	T205I	R203K, G204R	R203M	R203K, G204R	
	N	CAGACATTTTGCTCTCAAGCTG	R	**3′ end**	**S235F**					**M234I**
Charité Hospital Germany	N	GAGGAACGAGAAGAGGCTTG	R	**Both ends**						**A182S, S183Y, S186Y, S187L, S188L**
US CDC	N	GACCCCAAAATCAGCGAAAT	F	**3′ end**				Q9L *		
	N	ACCCCGCATTACGTTTGGTGGACC	P	5′ end					P13L	P13L, P13S
HKU Med Hong-Kong	N	CGAAGGTGTGACTTCCATG	R	5′ end				S327L *		
NIH Thailand	N	CCCCACTGCGTTCTCCATT	R	**Both ends**					**E31-R32-S33del**	
Chan CHINA	N	TTGGATCTTTGTCATCCAATTTG	R	**3′ end**					D343G*	
Young Singapore	S	TATACATGTCTCTGGGACCA	F	5′ end	H69del, V70del				H69del, V70del	H69del, V70del
	S	CTAAGAGGTTTGATAACCCTGTCCTACC	P	5’ end		D80A		K77T *		T76I, D80G
Chan China	S	CGCTCCAGGGCAAACTGGAAAG	P	**3′ end**		**K417N**	**K417T**	**Q414R**	**K417N**	**Q414K**
Sigma-Aldrich	S	AGACTAATTCTCCTCGGCGGGCACG	P	**Both ends**	**P681H**		**N679K**	**Q677H, P681R**	**N679K, P681H**	**Q677H, Ins679GIAL**
	S	CTAATTCTCCTCGGCGGGCACG	P	5′ end	P681H		N679K	Q677H, P681R	N679K, P681H	Q677H, Ins679GIAL
Charité Hospital Germany	RdRp	GTGARATGGTCATGTGTGGCGG	F	**Both ends**				**G671S**		**M666I, M666T**
Institut Pasteur FRANCE	RdRp	TCATACAAACCACGCCAGG	P	**3′ end**				**P227S**		**P227S**
***Qualitative PCR, and Sequencing assays***										
Sigma-Aldrich	N	GCCTCTTCTCGTTCCTCATCAC	F	**Both ends**						**A182S, S183Y, S186Y, S187L, S188L**
	N	AGCAGCATCACCGCCATTG	R	**5′ end**				G215C		G212C, G212V, N213Y, G214C
	N	AGCCTCTTCTCGTTCCTCATCAC	F	**Both ends**						**A182S, S183Y, S186Y, S187L, S188L**
	N	CCGCCATTGCCAGCCATTC	R	**Both ends**				G215C		**R209del, R209I, G212C, G212V, N213Y, G214C**
Won South Korea	S	CACCTGTGCCTGTTAAACCA	R	5′ end					T547K	
Thermo Fisher	S	GTGTTAATCTTACAACCAGAACTCAATTAC	F	**Both ends**		**L18F**	**L18F, T20N**	**T19R**	T19I, L24S, P25del*	**L18F**
Won South Korea	RdRp	CATGTGTGGCGGTTCACTAT	F	**3′ end**				**G671S**		

Mutations characterize various sublineages in SARS-CoV-2 variants of concern (VOC). The bold refers to mutations localized at 3’ end or both ends. * The asterisk refers to mutations that characterize subvariant or sublineages of the variant as fellow: the Q9L characterizes only Delta AY.43 sublineages, S327L only in Delta AY.5 sublineages, K77T only in Delta AY.35 and AY.48, D343G only in specific Omicron sublineage BA.1.15, while T19I, L24S, P25del only in Omicron BA.2 and its sublineages including the recently spotted recombinant XE. Abbreviations: F, forward primer; R, reverse primer; P, probe; N, nucleocapsid; S, spike; RdRp, polymerase.
